# Joint latent profiles of death anxiety and treatment adherence in HCC patients

**DOI:** 10.3389/fpsyt.2026.1792371

**Published:** 2026-05-11

**Authors:** Shoupeng Sheng, Lipeng Bai, Zhaxi Yundan, Hongwei Chen, Basang ZhaXi, LuoBu Duoji, Yi Shu, Xiaoqing Cao

**Affiliations:** 1 Center for Interventional Oncology, Beijing Youan Hospital, Capital Medical University, Beijing, China; 2 Senior Department of Traditional Chinese Medicine, Chinese PLA General Hospital, Beijing, China; 3 Hepato-Pancreato-Biliary Surgery, Lhasa People’s Hospital, Lhasa, Tibet Autonomous Region, China; 4 Department of General Surgery, Lhasa People’s Hospital, Lhasa, Tibet Autonomous Region, China; 5 Shenzhen Bao’an Authentic TCM Therapy Hospital, Guangzhou University of Chinese Medicine, Shenzhen, China; 6 Department of Thoracic Surgery, Beijing Chest Hospital, Capital Medical University, Beijing Tuberculosis and Thoracic Tumor Research Institute, Beijing, China

**Keywords:** death anxiety, fear of progression, health literacy, hepatocellular carcinoma, latent profile analysis, treatment adherence

## Abstract

**Background:**

Hepatocellular carcinoma (HCC), one of the leading contributors to the global cancer burden, often places patients in a dual predicament of pronounced death anxiety and suboptimal treatment adherence. Prior research has largely treated death anxiety and adherence as independent, homogeneous constructs, thereby overlooking potential within-population heterogeneity and their co-occurring patterns. This study adopted a person-centered approach to identify joint latent profiles of death anxiety and treatment adherence among patients with HCC and to examine factors associated with profile membership.

**Methods:**

A cross-sectional design was employed. From October to November 2025, 586 patients with HCC were recruited via convenience sampling from five tertiary general hospitals in Shenzhen, Beijing and Lhasa, China. Data were collected using the Death Anxiety Scale (DAS), General Medication Adherence Scale (GMAS), Health Literacy Scale Short-Form (HLS-SF), and Fear of Progression Questionnaire–Short Form (FoP-Q-SF).

**Results:**

Latent profile analysis identified three qualitatively distinct subgroups: low death anxiety–high treatment adherence, moderate death anxiety–moderate treatment adherence, and high death anxiety–low treatment adherence. Across profiles, death anxiety and treatment adherence exhibited a clear inverse co-variation pattern. Multinomial logistic regression indicated that health literacy and fear of disease progression were key psychosocial factors differentiating profile membership. In addition, demographic and disease-related variables showed varying predictive effects on profile assignment.

**Conclusions:**

Patients in the high death anxiety–low treatment adherence profile may represent a clinically important high-risk subgroup for targeted screening and supportive care. Clinical practice should emphasize assessment of health literacy and profile-specific psychosocial needs when planning stratified interventions. However, because of the cross-sectional design, the observed associations should not be interpreted causally, and longitudinal studies are needed to examine temporal transitions between profiles and their effects on subsequent treatment outcomes.

## 1 Introduction

Hepatocellular carcinoma (HCC) is among the malignancies with the heaviest disease burden worldwide (1, 2), characterized by high aggressiveness, late-stage presentation at diagnosis, and poor prognosis (3, 4). In 2020, there were approximately 906,000 new liver cancer cases globally and 830,000 deaths; China accounted for about 45.3% of all cases worldwide (5, 6). Given its insidious onset and rapid progression, most patients are diagnosed at intermediate to advanced stages, and long-term survival remains unsatisfactory (7). Beyond the substantial physical burden, patients with HCC frequently experience persistent and multifaceted psychological distress, among which death anxiety—directly tied to existential threat—warrants particular attention (8). Death anxiety generally refers to a sustained negative emotional experience, along with associated cognitive and behavioral responses, that arises when individuals recognize the finitude of life or encounter death-related cues or imminent threats (9). From the perspective of existential psychotherapy, confronting life’s limits renders death anxiety not merely an affective reaction but a force that can profoundly shape meaning-making, risk appraisal, and coping (10, 11). For patients with HCC, the cancer’s high lethality, uncertainty during treatment, fluctuations in therapeutic response and expectations, and risks of recurrence and progression may all intensify the perception of death-related threat (12, 13). Previous studies have reported that death anxiety in patients with HCC is significantly higher than in patients with other cancer types and is closely associated with cancer stage, time since diagnosis, and disease duration (14, 15). Nevertheless, much of the existing literature conceptualizes death anxiety as a unitary, homogeneous construct, neglecting potential heterogeneity within the patient population (16). This monolithic perspective limits a deeper understanding of the nature of death anxiety in HCC.

Treatment adherence refers to the extent to which patients’ behaviors—such as medication taking, follow-up attendance, lifestyle modification, and implementation of prescribed treatment plans—align with medical recommendations (17, 18). Within the comprehensive treatment framework for HCC, whether patients undergo surgical resection, transarterial chemoembolization, targeted therapy, or immune checkpoint inhibition, they typically face long-term, multi-stage regimens with dynamically adjusted treatment and follow-up schedules (19, 20). Adherence is therefore foundational to achieving therapeutic benefit, managing complications, and improving survival outcomes (21, 22). However, treatment adherence in clinical practice is often suboptimal among patients with HCC (23, 24). Poor adherence may lead to treatment interruption, loss to follow-up, delayed disease assessment, and inefficient use of healthcare resources, ultimately undermining quality of life and prognosis (25, 26). Accordingly, systematically elucidating the psychological and behavioral mechanisms underlying adherence variability and identifying high-risk subgroups are of substantial clinical value for developing precision interventions.

Importantly, the relationship between death anxiety and treatment adherence may be non-linear or context dependent. Terror management theory posits that death anxiety may activate defensive processes that promote avoidance, reducing engagement with threatening information or fostering passive coping, thereby weakening treatment implementation and patient–provider communication (27, 28). Conversely, the health belief model suggests that when perceived severity and susceptibility increase and actionable cues are available, threat perception may be transformed into motivation for health-promoting behaviors (29, 30). These seemingly divergent theoretical predictions imply that the effect of death anxiety on adherence is unlikely to be simply “the higher, the worse” or “the higher, the better”; rather, it may depend on patients’ available resources, cognitive appraisals, emotion-regulation capacity, and healthcare context (31). More crucially, patients’ levels of death anxiety and treatment adherence may form specific combinatorial patterns, yielding clinically meaningful subgroups. This premise provides a theoretical rationale for adopting a person-centered analytic framework.

Latent profile analysis (LPA) is a person-centered statistical approach that identifies unobserved, qualitatively distinct subgroups within a sample based on multiple continuous indicators, thereby uncovering hidden heterogeneity (32). Unlike variable-centered methods, LPA does not assume population homogeneity and instead emphasizes joint patterns across dimensions and clustering at the individual level (33). In recent years, LPA has been widely used to subtype psychological states and behaviors in health-related populations—for example, resilience profiles in patients with breast cancer (34), latent classes of adolescent internet addiction (35), and work–family balance profiles among nurses (36)—supporting the presence of substantial psycho-behavioral heterogeneity within specific groups. However, research that simultaneously incorporates death anxiety and treatment adherence within the same analytic framework to examine their joint profiles among patients with HCC remains limited. Prior studies have shown that patients with HCC can be differentiated into distinct subgroups based on tumor characteristics, multi-omics features, treatment-response patterns, or survival risk, underscoring that HCC is not a homogeneous condition at either the biological or clinical level (37–39). These precedents support the relevance of subgroup-oriented approaches in HCC research, but they have focused primarily on disease progression and outcome prediction rather than on psycho-behavioral heterogeneity. Accordingly, the present study extends this heterogeneity framework from prognostic and disease-centered stratification to the joint psychological and behavioral domain, specifically by identifying latent profiles characterized by death anxiety and treatment adherence.

The existing literature suggests that demographic characteristics, disease-related factors, and psychosocial resources may play important roles in shaping joint patterns of death anxiety and treatment adherence among patients with HCC. Demographic variables—such as age, sex, marital status, education, monthly income, employment, and place of residence—are closely linked to patients’ illness cognitions and coping choices (40, 41). Evidence indicates that older adults may hold different attitudes toward death due to life-stage differences (42, 43), whereas educational attainment and socioeconomic status may influence treatment behaviors by shaping patients’ ability to access and understand health information (44). Regarding disease characteristics, cancer stage directly reflects severity and prognosis, and patients with advanced-stage disease often face stronger perceptions of death-related threat (45, 46). Time since diagnosis and disease duration may influence emotional states and behavioral responses through processes of psychological adaptation (47, 48); prior studies suggest that psychological stress responses are most intense shortly after diagnosis and gradually shift toward adaptation over time (49).

Health literacy and fear of progression, as key psychosocial variables, may be particularly important for explaining the interplay between death anxiety and treatment adherence in HCC. Health literacy refers to individuals’ capacity to obtain, understand, appraise, and apply health information to make appropriate health decisions (50, 51). It directly influences how patients process illness-related information and their ability to comprehend and execute treatment plans (52). Studies have shown that higher health literacy can help patients more accurately interpret prognostic information—thereby reducing irrational death-related fear—while also facilitating effective communication with healthcare professionals and improving adherence (53, 54). Fear of progression refers to persistent worry that the disease may recur, metastasize, or worsen, and it is especially prevalent among patients with HCC (55, 56). Fear of progression is conceptually related to, yet distinct from, death anxiety: the former focuses on uncertainty in the disease course (55), whereas the latter is more explicitly oriented toward the ultimate issue of life’s end (57). These constructs may mutually reinforce each other and jointly influence psychological adjustment and treatment behavior. Nevertheless, how these factors operate in tandem to determine subgroup membership—namely, which variables effectively predict assignment to specific death anxiety–adherence profiles—has not been systematically examined in empirical research.

Addressing these theoretical and empirical gaps, this study used latent profile analysis to identify joint latent classes of death anxiety and treatment adherence among patients with HCC and further examined factors associated with different profile types. The innovations of this study are threefold. First, it integrates death anxiety and treatment adherence within a unified analytic framework, moving beyond prior work focused on single variables or bivariate associations to reveal their joint heterogeneous patterns. Second, by adopting a person-centered approach, it identifies clinically meaningful patient subgroups and provides actionable targets for precision psychological interventions. Third, it systematically evaluates the predictive roles of demographic characteristics, disease-related factors, and psychosocial variables in profile membership, offering evidence to support tailored, stratified management strategies. These findings may not only deepen theoretical understanding of psycho-behavioral interactions in HCC but also provide scientific guidance for psychological assessment and intervention in clinical practice.

## 2 Methods

### 2.1 Study design

This study employed a cross-sectional design. The LPA was used to identify joint latent classes characterized by death anxiety and treatment adherence among patients with liver cancer, and to further examine factors associated with different profile types. Owing to the cross-sectional nature of the data, the study was designed to detect between-profile heterogeneity and correlates of membership rather than to establish causal relationships or profile transitions over time.

### 2.2 Participants

#### 2.2.1 Participant Recruitment

From October to November 2025, patients with HCC were recruited using convenience sampling from the Departments of Hepatobiliary Surgery, Medical Oncology, and Interventional Radiology at five tertiary Grade A general hospitals in Shenzhen, Beijing, Lhasa, China. Because participants were recruited by convenience sampling from tertiary hospitals, the final sample is more appropriately interpreted as a tertiary-care hospital-based HCC cohort rather than a population-representative sample of all patients with HCC in China.

Prior to formal data collection, the research team contacted the nursing departments and heads of the relevant clinical units to provide a detailed description of the study objectives, procedures, ethics approval, and questionnaire administration. Consensus was reached regarding recruitment procedures and quality control measures. With departmental support, eligible inpatients were approached face-to-face in the wards by trained researchers, and eligible patients attending outpatient follow-up visits were also invited to participate. Before completing the questionnaire, all participants received a comprehensive explanation of informed consent. Researchers explained the study purpose, content, expected duration, potential risks and benefits in plain language and emphasized voluntariness and the right to withdraw at any time. Only patients who fully understood the information and voluntarily signed written informed consent were enrolled. Upon completion, questionnaires were checked on-site for completeness, and participants were promptly asked to fill in any missing items.

#### 2.2.2 Inclusion and exclusion criteria

To enhance methodological rigor, strict inclusion and exclusion criteria were applied.

Inclusion criteria were as follows: (1) age ≥18 years; (2) diagnosis of primary hepatocellular carcinoma (HCC) confirmed by histopathology (liver biopsy or surgical specimen) or clinical imaging; (3) awareness of their liver cancer diagnosis; (4) clear consciousness with adequate language comprehension and expression, enabling valid questionnaire responses; and (5) voluntary participation with provision of written informed consent.

Exclusion criteria were: (1) concomitant other primary malignancies, or secondary (metastatic) liver cancer; (2) a prior or current diagnosis of severe psychiatric disorders confirmed by a psychiatrist, or cognitive impairment confirmed by a neurologist; (3) terminal stage/end-of-life status with an estimated survival time <3 months as assessed by the attending physician; and (4) current participation in other interventional clinical studies that could affect psychological assessment outcomes.

#### 2.2.3 Minimum sample size

For LPA, Monte Carlo simulation studies suggest that, to ensure stable model estimation and reliable classification, the minimum sample size should be at least 500 (58). Nylund and colleagues further indicated that model-fit indices perform with relatively high accuracy in selecting the correct number of profiles when the sample size is ≥500 (59).

For the multinomial logistic regression analysis, an *a priori* sample size calculation was conducted using G*Power 3.1.9.7. Based on the planned inclusion of 12 primary predictors, a medium effect size (f² = 0.15), power (1−β) = 0.95, and α = 0.05, the estimated minimum required sample size was 184.

Considering both requirements, the larger value was adopted as the target sample size. Accounting for potential data loss due to incomplete questionnaires, poor response quality, or withdrawal, the planned recruitment target was set at 560 participants.

#### 2.2.4 Study sample

A total of 586 eligible patients with liver cancer were ultimately included. During data collection, 620 questionnaires were distributed and 586 valid questionnaires were returned, yielding an effective response rate of 94.52%. Among the 34 excluded questionnaires, 19 were from patients in the terminal stage, and 15 were from patients with other primary malignancies or secondary liver cancer. In addition, 12 questionnaires were excluded due to substantial missing data (>10% missing items), 15 were excluded due to obvious patterned responding (e.g., selecting the same option for more than 15 consecutive items), and 7 were excluded because the participant withdrew before completion. The final sample size (n = 586) exceeded the prespecified target (n = 560) and met the statistical requirements for both LPA and multinomial logistic regression.

#### 2.2.5 Ethical considerations

This study adhered to the ethical principles of the Declaration of Helsinki. The study protocol was approved by the Medical Ethics Committee of Shenzhen Bao’an Authentic TCM Therapy Hospital (No.: 2025-006K-02PJ). Written informed consent was obtained from all participants. Questionnaires were anonymized using coded identifiers, and strict confidentiality of all data was ensured.

### 2.3 Measures tools

#### 2.3.1 Death anxiety scale

Death anxiety was assessed using the Chinese version of the Death Anxiety Scale developed by Cai, Tang (60). The scale comprises 17 items across four domains: Dysphoria (5 items), Death Intrusion (5 items), Fear of Death (4 items), and Avoidance of Death (3 items). The instrument has been widely used in Chinese populations (61, 62), supporting its cultural applicability and reliability. Items are rated on a 5-point Likert scale, yielding a total score ranging from 17 to 85; higher scores indicate greater death anxiety. In this study, Cronbach’s α was 0.918, indicating excellent internal consistency. Confirmatory factor analysis (AMOS 30.0) demonstrated acceptable model fit: χ²/df = 3.670, GFI = 0.906, AGFI = 0.879, RMSEA = 0.068, CFI = 0.870, and TLI = 0.851.

#### 2.3.2 Treatment adherence scale

Treatment adherence was measured using the Chinese version of the General Medication Adherence Scale developed by Wang, Wang (63). The scale includes 11 items across three domains: Non-adherence due to patient behavior (5 items), Additional disease and pill burden (4 items), and Cost-related non-adherence (2 items). The scale has been applied in multiple Chinese patient populations, including older adults with stroke (64) and patients with chronic obstructive pulmonary disease (65), supporting its cultural suitability and reliability. Items are rated on a 5-point Likert scale, with total scores ranging from 11 to 55. Although the subscales are named as non-adherence domains, items were coded such that higher scores indicate better adherence. In this study, Cronbach’s α was 0.900. CFA results (AMOS 30.0) indicated acceptable model fit: χ²/df = 4.133, GFI = 0.938, AGFI = 0.907, RMSEA = 0.073, CFI = 0.902, and TLI = 0.877.

#### 2.3.3 Health literacy scale

Health literacy was assessed using the Chinese short form of the Health Literacy Scale developed by Sun, Lv (66). The scale consists of 12 items across three domains: Health care (4 items), Disease prevention (4 items), and Health promotion (4 items). It has been used in Chinese populations such as university students (67) and patients with colorectal cancer (68), demonstrating good cultural applicability and reliability. Items are rated on a 5-point Likert scale, with total scores ranging from 12 to 60; higher scores indicate higher health literacy. In this study, Cronbach’s α was 0.831. CFA (AMOS 30.0) indicated acceptable model fit: χ²/df = 3.568, GFI = 0.954, AGFI = 0.921, RMSEA = 0.066, CFI = 0.918, and TLI = 0.880.

#### 2.3.4 Fear of progression questionnaire

Fear of disease progression was measured using the Fear of Progression Questionnaire—Short Form (FoP-Q-SF) developed by Mahendran, Liu (69). The scale includes 12 items covering domains of affective reactions, partner/family, work, and loss of autonomy. The instrument was originally applied among Chinese patients with cancer, supporting its applicability in this context. Items are rated on a 5-point Likert scale, with total scores ranging from 12 to 60; higher scores indicate greater fear of progression. In this study, Cronbach’s α was 0.814. CFA (AMOS 30.0) indicated excellent model fit: χ²/df = 1.091, GFI = 0.987, AGFI = 0.976, RMSEA = 0.012, CFI = 0.997, and TLI = 0.995.

### 2.4 Statistical analysis

All analyses were performed using SPSS 27.0, AMOS 30.0, and Mplus 8.3. All statistical tests were two-sided, with α = 0.05. Scale reliability was evaluated using Cronbach’s α (α > 0.70 considered acceptable). Structural validity was examined using confirmatory factor analysis (CFA). Model fit was assessed using χ²/df (<5 acceptable), the comparative fit index (CFI ≥ 0.80), Tucker–Lewis index (TLI ≥ 0.80), and the root mean square error of approximation (RMSEA ≤ 0.08).

Common method bias was examined using Harman’s single-factor test, with 40% as the threshold. Continuous variables are presented as mean ± standard deviation (M ± SD), and categorical variables as frequency and percentage. Pearson correlation analysis was used to examine associations among continuous variables (70). LPA was conducted to identify latent subgroups based on death anxiety and treatment adherence. Models were estimated sequentially from one to five profiles. After selecting the optimal profile solution, chi-square tests were used to compare categorical demographic and disease-related characteristics across profiles, and one-way ANOVA was used to compare continuous psychosocial variables (health literacy and fear of progression) between profiles. When the omnibus ANOVA was significant, Fisher’s least significant difference (LSD) test was used for *post-hoc* pairwise comparisons. Profile membership was treated as the dependent variable. Variables significant in univariate analyses (p < 0.05) were entered into a multinomial logistic regression model using forward stepwise selection to examine the effects of demographic characteristics, disease-related factors, and psychosocial variables on profile membership.

## 3 Results

### 3.1 Common method bias

Although this study incorporated reverse-worded items and emphasized confidentiality and anonymity, common method bias may still be present because all variables were collected via self-report. Therefore, we conducted Harman’s single-factor test by performing an unrotated exploratory factor analysis on all measurement items. The first factor accounted for 18.961% of the total variance, which is well below the conventional 40% threshold. These findings indicate that common method bias is unlikely to be a serious concern in the present dataset. Nevertheless, because all core variables were obtained through self-report, shared method variance, recall bias, and social desirability effects cannot be fully excluded.

### 3.2 Sociodemographic characteristics

A total of 586 patients with hepatocellular carcinoma (HCC) were included. Regarding gender distribution, 405 patients were male (69.1%) and 181 were female (30.9%). Participants were aged 25–85 years, with a mean age of 54.72 ± 9.689 years. The largest proportion had a junior high school education (n = 207, 35.3%); 341 participants (58.2%) resided in urban areas, and most were married (n = 396, 67.6%). For disease-related characteristics, according to the Barcelona Clinic Liver Cancer (BCLC) staging system (71), stage C was the most common (n = 150, 25.6%), suggesting a relatively high proportion of intermediate-to-advanced cases in this sample. Time since diagnosis was predominantly within 3 months (n = 186, 31.7%). Additional details are shown in **Table 1**.

**Table 1:** Sociodemographic characteristics of the study sample.

Variables	Items	Frequency (N)	Proportion (%)
Gender	Male	405	69.1%
	Female	181	30.9%
Education background	Primary school and below	118	20.1%
	Junior high school	207	35.3%
	High school	167	28.5%
	College or above	94	16.0%
Residence	Cities	341	58.2%
	Countryside	245	41.8%
Marital status	Divorce	63	10.8%
	Widowed	27	4.6%
	Unmarried	100	17.1%
	Married	396	67.6%
Monthly income level	3000 RMB or less	250	42.7%
	3001–6000 RMB	181	30.9%
	6001–9000 RMB	86	14.7%
	9001 RMB and above	69	11.8%
Working condition	Unemployment	94	16.0%
	Retirement	168	28.7%
	Student	43	7.3%
	Staff and workers	281	48.0%
Cancer stage	Stage 0	63	10.8%
	Stage A	96	16.4%
	Stage B	140	23.9%
	Stage C	150	25.6%
	Stage D	137	23.4%
Time since diagnosis	Within three months	186	31.7%
	Within 3 to 6 months	159	27.1%
	Within 6–12 months	143	24.4%
	1 year ago	98	16.7%
Age (year)		54.72 ± 9.689	

### 3.3 Descriptive statistics and correlation analysis

Descriptive statistics and correlation results are summarized in **Table 2**. Skewness ranged from −0.343 to 0.324 and kurtosis ranged from −0.873 to 4.475. According to the criteria proposed by Kline (72), these values indicate that the data meet the assumption of approximate normality.

**Table 2:** Descriptive statistics and correlations among study variables.

Variables	M	SD	Skewness	Kurtosis	1	2	3	4
Death anxiety	3.026	0.512	-0.022	-0.873	1			
Treatment adherence	2.977	0.553	-0.033	-0.594	-0.676***	1		
Health literacy	3.164	0.489	-0.343	4.475	-0.308***	0.087*	1	
Fear of disease progression	3.020	0.469	0.324	3.533	0.560***	-0.440***	-0.138***	1

***p<0.001; *p < 0.05.

Correlation analyses showed that death anxiety was strongly and negatively correlated with treatment adherence (r = −0.676, p < 0.001), suggesting that elevated death anxiety may lead to avoidance, denial, or psychological decompensation, thereby reducing adherence to treatment. Death anxiety was moderately and negatively correlated with health literacy (r = −0.308, p < 0.001) and strongly and positively correlated with fear of disease progression (r = 0.560, p < 0.001). Treatment adherence was weakly positively correlated with health literacy (r = 0.087, p < 0.05) and moderately negatively correlated with fear of disease progression (r = −0.440, p < 0.001). Health literacy was weakly and negatively correlated with fear of disease progression (r = −0.138, p < 0.001). Overall, these correlations provide preliminary evidence for the pattern of associations among the study variables.

### 3.4 Latent profile analysis

#### 3.4.1 Identification of the optimal profile solution

Using the four dimension scores of the Death Anxiety Scale and the three dimension scores of the Treatment Adherence Scale as observed indicators, latent profile models with one to five profiles were estimated. Model fit indices are presented in **Table 3**. As the number of profiles increased from 1 to 5, AIC, BIC, and sample-size adjusted BIC (aBIC) decreased monotonically, indicating improved fit with additional profiles. The Lo–Mendell–Rubin (LMR) test and the bootstrap likelihood ratio test (BLRT) were significant for the 2- to 4-profile solutions (all p < 0.05), suggesting that these models fit significantly better than the corresponding models with one fewer profile. However, the LMR test for the 5-profile solution was not significant (p = 0.525), indicating no significant improvement over the 4-profile model.

**Table 3:** Fit indices for the 1–5 profile latent profile models.

Profile	AIC	BIC	aBIC	Entropy	LMR (p)	BLRT (p)	Proportion of potential subgroups
1	8358.379	8419.606	8375.161	–	–	–	–
2	7181.369	7277.582	7207.740	0.803	<0.001	<0.001	48.9%/51.1%
3	6860.738	7011.938	6916.698	0.835	<0.001	<0.001	29.8%/39.0%/31.2%
4	6796.432	6962.619	6841.982	0.878	0.023	<0.001	1.0%/28.9%/39.6%/30.5%
5	6761.168	6962.340	6816.306	0.897	0.525	<0.001	39.1%/1.0%/28.8%/30.1%/0.9%

Further comparison between the 3- and 4-profile solutions showed that although the 4-profile model yielded a significant LMR test (p = 0.023), its smallest profile comprised only 1.0% of the sample, far below the commonly recommended 5% minimum, raising concerns about overextraction and limited clinical interpretability of this very small class. In contrast, the 3-profile model demonstrated satisfactory performance across indices (AIC = 6860.738, BIC = 7011.938, aBIC = 6916.698), with an entropy of 0.835 (indicating >90% classification accuracy). Both LMR and BLRT were highly significant (p < 0.001), and class proportions were 29.8%, 39.0%, and 31.2%, respectively—each exceeding the 5% criterion and relatively well balanced. Accordingly, the 3-profile solution was selected as the optimal model.

#### 3.4.2 Profile labeling and characterization

**Figure 1:**
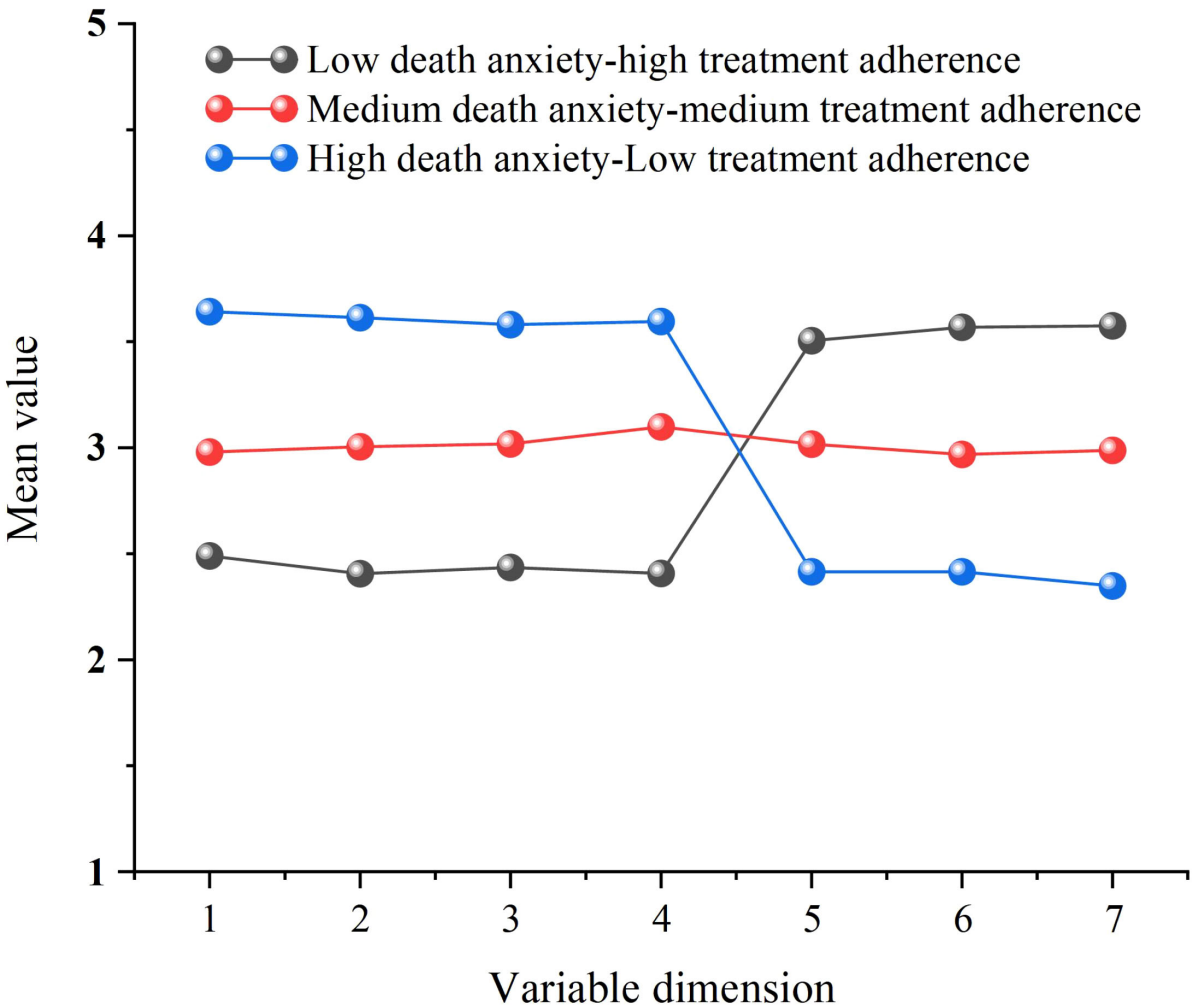
Score patterns for each dimension of the three latent profiles of death anxiety and treatment adherence. 1, Dysphoria; 2, Death Intrusion; 3, Fear of Death; 4, Avoidance of Death; 5, Non-adherence due to patient behavior; 6, Additional disease and pill burden; 7, Cost-related non-adherence.

The patterns of scores across dimensions of death anxiety and treatment adherence for the three latent profiles are displayed in **Figure 1**.

Profile 1: Low death anxiety–high treatment adherence (LDA–HTA). This profile included 175 patients (29.8% of the total sample). The pattern suggests good psychological adaptation, with relatively mild anxiety responses to illness- and death-related concerns and active cooperation with treatment recommendations.

Profile 2: Moderate death anxiety–moderate treatment adherence (MDA–MTA). This profile included 228 patients (39.0%), representing the largest subgroup. This pattern indicates a transitional state of psychological adjustment, characterized by a moderate level of death-related anxiety that does not yet severely compromise adherence behavior.

Profile 3: High death anxiety–low treatment adherence (HDA–LTA). This profile included 183 patients (31.2%). The pattern reflects substantial death anxiety accompanied by pronounced barriers to implementing treatment, identifying a high-risk subgroup requiring targeted attention and intervention.

Across profiles, death anxiety and treatment adherence exhibited a clear negative co-variation: profiles with higher death anxiety consistently showed lower treatment adherence. This finding corroborates the correlation results and further indicates distinct subgroup differentiation at the individual level.

### 3.5 Differences between latent profiles in sociodemographic, clinical, and psychosocial variables

Chi-square tests and one-way ANOVA were used to compare the three latent profiles on sociodemographic characteristics, disease-related characteristics, and psychosocial variables. For psychosocial variables with significant omnibus ANOVA results, LSD *post-hoc* pairwise comparisons were conducted. Results are presented in **Table 4**.

**Table 4:** Comparisons of sociodemographic, clinical, and psychosocial characteristics across the three latent profiles.

Variables	Items	Profile 1	Profile 2	Profile 3	χ²/F	p
Gender	Male	120	144	141	9.211	0.010
	Female	55	84	42		
Education background	Primary school and below	32	44	42	2.42	0.877
	Junior high school	63	77	67		
	High school	51	69	47		
	College or above	29	38	27		
Residence	Cities	88	134	119	8.042	0.018
	Countryside	87	94	64		
Marital status	Divorce	28	20	15	14.012	0.029
	Widowed	11	11	5		
	Unmarried	35	37	28		
	Married	101	160	135		
Monthly income level	3000 RMB or less	80	92	78	14.435	0.025
	3001–6000 RMB	38	79	64		
	6001–9000 RMB	29	37	20		
	9001 RMB and above	28	20	21		
Working condition	Unemployment	23	38	33	6.852	0.335
	Retirement	55	69	44		
	Student	16	11	16		
	Staff and workers	81	110	90		
Cancer stage	Stage 0	30	22	11	19.343	0.013
	Stage A	19	43	34		
	Stage B	40	49	51		
	Stage C	44	65	41		
	Stage D	42	49	46		
Time since diagnosis	Within three months	59	77	50	14.088	0.029
	Within 3 to 6 months	35	67	57		
	Within 6–12 months	41	50	52		
	1 year ago	40	34	24		
Age		54.59 ± 9.733	54.89 ± 9.326	54.63 ± 10.131	0.061	0.941
Health literacy		3.265 ± 0.524 ^a^	3.147 ± 0.416 ^b^	3.089 ± 0.526 ^b^	6.139	0.002
Fear of disease progression		3.097 ± 0.544 ^a^	3.019 ± 0.380 ^ab^	2.948 ± 0.484 ^b^	4.568	0.011

Profile 1: Low death anxiety–high treatment adherence (LDA–HTA); Profile 2: Moderate death anxiety–moderate treatment adherence (MDA–MTA); Profile 3: High death anxiety–low treatment adherence (HDA–LTA). Group differences were tested using one-way ANOVA. When the omnibus ANOVA was significant, Fisher’s least significant difference (LSD) test was used for post-hoc pairwise comparisons. Values with different superscript letters indicate significant differences between profiles at.

p<0.05; values sharing at least one superscript letter do not differ significantly.

Profile Differences in Sociodemographic Characteristics. Gender distribution differed significantly across the three profiles (χ² = 9.211, p = 0.010). The proportion of females was 31.4% in Profile 1 (LDA–HTA), 36.8% in Profile 2 (MDA–MTA), and only 22.9% in Profile 3 (HDA–LTA). This suggests that male patients were relatively overrepresented in the high death anxiety–low adherence subgroup.

Marital status (χ² = 14.012, p = 0.029), monthly income level (χ² = 14.435, p = 0.025), place of residence (χ² = 8.042, p = 0.018) differed significantly across profiles. No significant differences were observed in age (F = 0.061, p = 0.941) or educational level (χ² = 2.420, p = 0.877). Employment status was also not statistically significant across profiles (χ² = 6.852, p = 0.335).

Profile Differences in Disease-Related Characteristics. Cancer stage (χ² = 19.343, p = 0.013) and time since diagnosis (χ² = 14.088, p = 0.029), differed significantly across profiles.

Health literacy differed significantly across profiles (F = 6.139, p = 0.002). Fisher’s LSD *post-hoc* comparisons showed that health literacy was significantly higher in Profile 1 (3.265 ± 0.524) than in Profile 2 (3.147 ± 0.416, p = 0.016) and Profile 3 (3.089 ± 0.526, p < 0.001), whereas the difference between Profiles 2 and 3 was not statistically significant (p = 0.225). Fear of disease progression also differed significantly across profiles (F = 4.568, p = 0.011). *Post-hoc* comparisons indicated that Profile 1 (3.097 ± 0.544) had significantly higher fear of disease progression than Profile 3 (2.948 ± 0.484, p = 0.003), whereas the differences between Profile 1 and Profile 2 (p = 0.098) and between Profile 2 and Profile 3 (p = 0.124) were not statistically significant. This suggests that although patients in the low death anxiety–high adherence subgroup reported lower death anxiety, their concerns about disease progression were not correspondingly reduced and were, in fact, slightly higher than those in the other two groups.

### 3.6 Multinomial logistic regression for latent profile membership

To further identify factors associated with latent profile membership, a multinomial logistic regression was performed using Profile 1 as the reference group. Variables showing statistically significant differences in the univariate analyses were entered as predictors. Results are shown in **Table 5**.

**Table 5:** Multinomial logistic regression analysis of factors associated with latent profile membership.

Profile	Variables	Items	B	SE	Wald χ²	P	OR	95% CI
Profile 2	Health literacy		-0.574	0.235	5.960	0.015	0.563	0.356~0.893
	Fear of disease progression		-0.503	0.233	4.656	0.031	0.605	0.383~0.955
	Gender	Male	-0.321	0.226	2.005	0.157	0.726	0.466~1.131
		Female (refer)						
	Marital status	Divorce	-0.833	0.337	6.121	0.013	0.435	0.225~0.841
		Widowed	-0.565	0.462	1.500	0.221	0.568	0.230~1.404
		Unmarried	-0.296	0.282	1.103	0.294	0.744	0.428~1.292
		Married (refer)						
	Residence	Cities	0.325	0.213	2.340	0.126	1.384	0.913~2.100
		Countryside (refer)						
	Working condition	Unemployment	0.322	0.316	1.039	0.308	1.379	0.743~2.560
		Retirement	-0.071	0.245	0.084	0.772	0.932	0.577~1.505
		Student	-0.766	0.434	3.117	0.077	0.465	0.199~1.088
		Staff and workers (refer)						
	Cancer stage	Stage 0	-0.285	0.369	0.596	0.440	0.752	0.365~1.550
		Stage A	0.809	0.361	5.011	0.025	2.245	1.106~4.558
		Stage B	0.217	0.313	0.482	0.487	1.243	0.673~2.294
		Stage C	0.326	0.302	1.163	0.281	1.385	0.766~2.504
		Stage D (refer)						
	Time since diagnosis	Within three months	-0.300	0.329	0.833	0.361	0.741	0.389~1.411
		Within 3 to 6 months	0.435	0.308	1.991	0.158	1.545	0.844~2.826
		Within 6–12 months	0.073	0.282	0.067	0.796	1.076	0.619~1.870
		1 year ago (refer)						
Profile 3	Health literacy		-0.803	0.252	10.152	0.001	0.448	0.273~0.734
	Fear of disease progression		-0.948	0.263	13.022	0.001	0.388	0.232~0.649
	Gender	Male	0.357	0.257	1.923	0.166	1.429	0.863~2.365
		Female (refer)						
	Marital status	Divorce	-1.063	0.379	7.882	0.005	0.346	0.165~0.726
		Widowed	-1.073	0.580	3.425	0.064	0.342	0.110~1.066
		Unmarried	-0.386	0.306	1.594	0.207	0.679	0.373~1.238
		Married (refer)						
	Residence	Cities	0.584	0.232	6.355	0.012	1.793	1.139~2.824
		Countryside (refer)						
	Working condition	Unemployment	0.466	0.335	1.941	0.164	1.594	0.827~3.072
		Retirement	-0.273	0.271	1.013	0.314	0.761	0.447~1.295
		Student	-0.197	0.421	0.219	0.640	0.821	0.359~1.875
		Staff and workers (refer)						
	Cancer stage	Stage 0	-1.019	0.441	5.328	0.021	0.361	0.152~0.857
		Stage A	0.593	0.380	2.433	0.119	1.809	0.859~3.811
		Stage B	0.348	0.322	1.167	0.280	1.417	0.753~2.665
		Stage C	-0.110	0.327	0.114	0.736	0.896	0.472~1.700
		Stage D (refer)						
	Time since diagnosis	Within three months	-0.580	0.354	2.676	0.102	0.560	0.280~1.122
		Within 3 to 6 months	0.217	0.319	0.460	0.498	1.242	0.664~2.322
		Within 6–12 months	-0.371	0.302	1.504	0.220	0.690	0.382~1.248
		1 year ago (refer)						

Profile 1 served as the reference category for the dependent variable. Regression coefficients are presented as B, with corresponding standard errors (SE), Wald χ2 statistics, odds ratios (OR), and 95% confidence intervals (CI).

#### 3.6.1 Profile 2 versus profile 1

Regarding psychosocial variables, health literacy was a significant predictor distinguishing Profile 2 from Profile 1 (OR = 0.563, 95% CI: 0.356–0.893, p = 0.015). This indicates that for each one-unit increase in health literacy, the odds of belonging to the moderate death anxiety–moderate treatment adherence group decreased by 43.7%, suggesting that higher health literacy serves as a protective factor for membership in the low death anxiety–high treatment adherence group. Fear of disease progression was also a significant predictor (OR = 0.605, 95% CI: 0.383–0.955, p = 0.031), such that lower fear of disease progression was associated with a higher likelihood of belonging to Profile 2 rather than Profile 1.

In terms of sociodemographic characteristics, marital status significantly predicted profile membership. Compared with married patients, divorced patients were significantly less likely to belong to Profile 2 (OR = 0.435, 95% CI: 0.225–0.841, p = 0.013), indicating that divorced patients were more likely to be classified into the low death anxiety–high treatment adherence group. Sex, place of residence, and employment status were not statistically significant in the Profile 2 versus Profile 1 comparison (all p > 0.05).

With respect to disease-related characteristics, patients with stage A disease were more likely to belong to Profile 2 than those with stage D disease (OR = 2.245, 95% CI: 1.106–4.558, p = 0.025). This finding suggests that, relative to patients with advanced-stage disease, those with stage A disease may be more likely to exhibit a transitional psychological adaptation pattern characterized by moderate levels of death anxiety and treatment adherence. Time since diagnosis did not significantly predict membership between these two profiles (p > 0.05).

#### 3.6.2 Profile 3 versus profile 1

Among psychosocial variables, health literacy showed an even stronger predictive effect for membership in Profile 3 (OR = 0.448, 95% CI: 0.273–0.734, p = 0.001). Specifically, each one-unit increase in health literacy was associated with a 55.2% reduction in the odds of belonging to the high death anxiety–low treatment adherence group. Compared with the Profile 2 versus Profile 1 contrast, the effect of health literacy was more pronounced in distinguishing Profile 3, underscoring its central role in identifying this high-risk subgroup. Fear of disease progression was likewise a significant predictor (OR = 0.388, 95% CI: 0.232–0.649, p < 0.001), with a stronger effect than that observed for Profile 2, indicating that lower fear of disease progression was associated with a greater likelihood of belonging to the high death anxiety–low adherence group.

Regarding sociodemographic characteristics, marital status remained a significant predictor in the Profile 3 versus Profile 1 comparison. Divorced patients were significantly less likely than married patients to belong to Profile 3 (OR = 0.346, 95% CI: 0.165–0.726, p = 0.005). Notably, place of residence reached statistical significance in this comparison (OR = 1.793, 95% CI: 1.139–2.824, p = 0.012), indicating that urban residents were more likely than rural residents to be classified into the high death anxiety–low treatment adherence group. Sex and employment status were not significant predictors (p > 0.05).

In terms of disease-related characteristics, patients with stage 0 disease were significantly less likely than those with stage D disease to belong to Profile 3 (OR = 0.361, 95% CI: 0.152–0.857, p = 0.021), suggesting that patients at an early stage are less likely to exhibit the high death anxiety–low adherence psycho-behavioral pattern. This further supports a close association between disease severity and patients’ psycho-behavioral profiles. Time since diagnosis was not a significant predictor in the Profile 3 versus Profile 1 comparison (p > 0.05).

The multinomial logistic regression results indicate that health literacy and fear of disease progression are core psychosocial factors differentiating the three latent profiles, with particularly pronounced effects observed in the Profile 3 versus Profile 1 comparison. Additionally, sociodemographic and clinical factors—especially marital status, place of residence, and cancer stage—exhibited varying predictive associations with profile membership, offering multidimensional indicators for identifying high-risk subgroups. These findings provide evidence to support stratified clinical management and precision-targeted interventions.

## Discussion

### 4.1 Latent profile characteristics of death anxiety and treatment adherence in HCC patients

Three qualitatively distinct latent profiles were identified: low death anxiety–high treatment adherence (29.8%), moderate death anxiety–moderate treatment adherence (39.0%), and high death anxiety–low treatment adherence (31.2%). This pattern corroborates substantial psycho-behavioral heterogeneity within the HCC population, extending prior work that has often treated death anxiety and adherence as homogeneous constructs. The relatively balanced distribution across profiles suggests that each psycho-behavioral pattern is meaningfully represented in clinical settings and warrants targeted attention.

Across the three profiles, death anxiety and treatment adherence demonstrated a pronounced negative covariation, consistent with the core assumptions of terror management theory. According to this theory, exposure to mortality threats may activate proximal defense mechanisms manifested as avoidance, denial, or psychological disengagement from threat-related information, thereby diminishing attention to—and willingness to enact—treatment-related recommendations. For patients in the high death anxiety–low adherence group, persistent and intense death anxiety may promote avoidant coping, reflected in reduced engagement with healthcare services, neglect of medical advice, or discontinuation of treatment. Although such defensive processes may transiently alleviate distress, they may ultimately compromise treatment effectiveness and survival outcomes over the long term.

Notably, the moderate death anxiety–moderate adherence group constituted the largest proportion of the sample (39.0%), with important clinical implications. These patients appear to be in a transitional state of psychological adaptation—neither the most optimal psycho-behavioral pattern nor the highest-risk state requiring urgent intervention. From the perspective of the Health Belief Model, a moderate level of perceived threat may exert dual effects: it can strengthen treatment motivation by increasing perceived severity, yet—if self-efficacy and coping resources are insufficient—may also generate hesitation and uncertainty that impede action. Accordingly, this subgroup may represent a critical “window of opportunity” for clinical intervention, wherein timely psychological support and health education could facilitate a shift toward a more adaptive psycho-behavioral pattern.

The presence of the low death anxiety–high adherence group suggests that, even when facing a life-threatening illness, some patients achieve relatively good psychological adjustment and sustain active participation in treatment. This may be attributable to multiple protective factors, such as effective emotion regulation, adequate social support, accurate illness/treatment cognitions, and high-quality communication with the healthcare team. The psycho-behavioral characteristics of this group provide an important reference for understanding resilience mechanisms in cancer populations, and its protective factors warrant further investigation.

### 4.2 Effects of psychosocial factors on latent profile membership

Health literacy and fear of disease progression emerged as the central psychosocial factors distinguishing the three profiles, with the strongest predictive effects observed when contrasting the high death anxiety–low adherence and low death anxiety–high adherence groups.

Health literacy showed a robust and graded association with profile membership. Lower health literacy significantly increased the likelihood of belonging to the moderate or high death anxiety profiles. Several mechanisms may underlie this pattern. Patients with limited health literacy may have more difficulty accurately understanding disease information, prognosis, treatment rationale, and side-effect management, which can increase uncertainty and reduce perceived control. Lower health literacy may also impede effective communication with clinicians and weaken patients’ ability to translate medical recommendations into concrete self-management behaviors. In this sense, health literacy may function not only as an informational resource but also as a psychological regulatory resource that helps patients cognitively organize illness-related threats and remain engaged in treatment.

The predictive pattern of fear of disease progression differed from expectations. Lower scores on fear of disease progression were associated with membership in the moderate or high death anxiety profiles. This pattern appears counterintuitive, because one might expect patients with greater death anxiety to report greater fear of progression as well. First, fear of progression and death anxiety are related but non-identical forms of threat processing. Fear of progression is primarily illness-focused and future-oriented; it concerns recurrence, metastasis, symptom worsening, functional decline, and the uncertainty of disease trajectory. Death anxiety, by contrast, refers to a broader existential response to finitude, mortality salience, and the possibility of non-being. Thus, fear of progression is centered on “what may happen to my disease,” whereas death anxiety is centered more fundamentally on “what it means that my life may end.” Although these two experiences may positively covary in many patients, they need not move in parallel once existential threat becomes dominant. Second, from a psychodynamic and existential perspective, lower fear of progression in the high death anxiety profile may reflect a shift in the level at which threat is mentally represented. When mortality concerns become highly salient, some patients may move beyond concrete worries about disease progression and become preoccupied with more global meanings of loss, finitude, helplessness, or annihilation. In such a state, the illness-specific question of whether the tumor will progress may become psychologically less central than the broader confrontation with death itself. Put differently, fear of progression may be attenuated not because the patient feels safe, but because the patient’s distress has migrated from the clinical trajectory of the disease to the existential horizon of mortality. Third, Terror management theory and related models of defensive responding suggest that overwhelming mortality awareness can activate avoidance, emotional numbing, disengagement from threatening information, or a form of resigned detachment. Under such conditions, patients may report lower illness-specific worries not because threat is absent, but because attention to specific progression-related concerns has been defensively reduced or collapsed into a more diffuse sense of existential dread. Clinically, this interpretation is consistent with the low adherence observed in Profile 3: if patients disengage from disease-specific monitoring and treatment-related information, they may simultaneously report less fear of progression while showing poorer behavioral engagement.

### 4.3 Effects of demographic and disease-related factors on latent profile membership

This study further examined demographic and disease-related predictors of profile membership. Multinomial logistic regression indicated that marital status, residence (urban vs. rural), and cancer stage significantly influenced latent profile membership among HCC patients.

Marital status exhibited a predictive pattern that diverged from conventional social support expectations. Compared with married patients, divorced patients were significantly less likely to belong to either the moderate death anxiety–moderate adherence group or the high death anxiety–low adherence group. This finding implies that the role of marriage in psychological adjustment among HCC patients may be dual-edged. On one hand, married patients may benefit from spousal emotional support and caregiving resources (73); on the other hand, they may experience heightened pressures arising from family responsibilities and role expectations. Given the poor prognosis often associated with HCC, concerns about no longer fulfilling family roles, worries about spouses’ and children’s future wellbeing, and the financial burden of treatment may intensify death anxiety. In contrast, divorced patients may lack direct spousal support but may also experience reduced role-related burden and psychological strain. Moreover, divorced individuals may develop greater self-efficacy and resilience through independent coping with life challenges, which could help maintain psychological stability when facing illness threats. Nevertheless, this interpretation requires confirmation through more granular assessments of family functioning, caregiving burden, and social support networks in future studies.

Residence primarily differentiated profile 3 versus profile 1. Urban residents were more likely than rural residents to belong to the high death anxiety–low adherence group. Urban patients typically have higher educational attainment and easier access to health information, which may yield a clearer understanding of HCC characteristics, treatment complexity, and prognosis. However, without sufficient psychological coping resources, accurate recognition of disease severity may exacerbate death anxiety. Furthermore, the fast-paced, highly competitive urban environment and relatively detached interpersonal relations may weaken the protective role of traditional social support networks. Thus, even within resource-rich medical contexts, urban patients may experience greater deficits in emotional support and psychological comfort. By contrast, tighter community ties, mutual aid traditions, and extended family structures in rural settings may provide more emotional and instrumental support, buffering the psychological impact of illness. Cultural differences between urban and rural contexts regarding illness coping and attitudes toward death may also contribute: rural patients may be more inclined toward acceptance-based coping, whereas urban patients may prefer problem-focused strategies—strategies that may provoke stronger feelings of lost control and anxiety when confronted with a prognostically uncertain disease.

Cancer stage—an objective indicator of disease severity—predicted profile membership in a clinically expected yet nuanced manner. In the comparison between profile 3 and profile 1, patients with stage 0 disease were significantly less likely than those with stage D disease to be classified in the high death anxiety–low adherence group. This finding reflects a straightforward association between disease severity and psychological state: early-stage patients face lower objective mortality threat, have more treatment options, and generally have better prognosis, and thus are more likely to maintain lower death anxiety and higher adherence. Conversely, advanced-stage patients confront limited treatment options, greater uncertainty in treatment response, and shortened life expectancy, which may jointly elevate death anxiety and reduce treatment motivation.

However, the predictive effect of stage differed when comparing profile 2 with profile 1. Patients with stage A disease were more likely than those with stage D disease to belong to the moderate death anxiety–moderate adherence group, indicating that early-stage patients do not necessarily manifest the most adaptive psycho-behavioral pattern. Although stage A represents relatively early disease, the psychological shock associated with a cancer diagnosis may not have fully subsided, and patients may remain in a transitional phase of adjustment. At this time, patients may simultaneously hope for therapeutic benefit while fearing progression or recurrence, producing moderate levels of death anxiety and adherence. Clinically, this underscores that early-stage patients’ psychological support needs should not be underestimated; timely assessment and intervention may promote progression toward a more adaptive psycho-behavioral pattern.

Notably, time since diagnosis did not reach statistical significance in the multinomial logistic regression, differing from the univariate findings. This discrepancy may reflect collinearity or interaction effects between time since diagnosis and other predictors; after controlling for variables such as health literacy and fear of disease progression, the independent contribution of time since diagnosis may have attenuated. Nonetheless, in light of univariate results and prior literature, the role of time since diagnosis in psychological adaptation should not be disregarded. The early post-diagnosis period (approximately 3–6 months) may represent a key window for psychological intervention, when needs are most acute and timely support may help prevent transitions into high-risk profiles.

Taken together, the effects of demographic and disease-related factors on joint death anxiety–adherence profiles are multidimensional and complex. The predictive role of marital status suggests that clinicians should attend to family responsibility-related stress among married patients rather than assuming marriage is uniformly protective. The residence effect highlights potential gaps in psychological support among urban patients, indicating the need for enhanced psychosocial attention to this group. Cancer stage provides an objective basis for early identification of high-risk subgroups while also reminding clinicians to address psychological adjustment needs even among early-stage patients. These findings provide important evidence to guide differentiated and precision-based psychological intervention strategies.

### 4.4 Theoretical and clinical implications

This study contributes meaningfully at both theoretical and practical levels. Theoretically, it extends subgroup-oriented research in HCC into the psycho-behavioral domain. Although prior HCC studies have used heterogeneity-based strategies to classify patients according to prognostic features, biological characteristics, or survival-related risk, these approaches have rarely been applied to psychological and behavioral processes (37–39). By placing death anxiety and treatment adherence within a unified person-centered framework, the present study moves beyond traditional variable-centered analyses and demonstrates that clinically meaningful psycho-behavioral profiles can also be identified within HCC populations. In this sense, our findings complement existing prognostic stratification work in HCC by showing that heterogeneity in this disease is not confined to tumor biology or survival outcomes, but is also reflected in patients’ psychological adaptation and treatment-related behavior.

Practically, the identified profiles provide an evidence base for stratified management and precision interventions in HCC. First, these three profiles can serve as a classification framework for psycho-behavioral assessment, enabling clinicians to rapidly identify high-risk subgroups. Patients in the high death anxiety–low adherence group should be prioritized for intervention and require coordinated multidisciplinary attention, including specialized support from psycho-oncology professionals, resource linkage by social workers, and strengthened communication by medical staff. Second, health literacy—an inherently modifiable protective factor—should be considered a key intervention target. For patients with low health literacy, simplified delivery of health information, visually supported educational materials, and individualized communication coaching may facilitate understanding of disease information and treatment plans. Third, the period from approximately 3 months to 1 year after diagnosis may represent an optimal timeframe for psychological intervention, during which proactive assessment and timely support should be provided to prevent progression toward the high death anxiety–low adherence pattern.

### 4.5 Limitations and future directions

Despite the above findings, several limitations should be acknowledged. First, the cross-sectional design precludes causal inference. The present data cannot determine whether death anxiety contributes to poorer treatment adherence, whether low adherence intensifies death-related distress, or whether both are shaped by shared upstream factors. In addition, profile stability and transitions over time could not be examined. Future studies should adopt longitudinal follow-up designs and, where possible, latent transition analysis to clarify temporal ordering, developmental trajectories, and movement between psycho-behavioral profiles. Second, participants were recruited by convenience sampling from five tertiary hospitals, and the sample should therefore be interpreted as a tertiary-care hospital-based cohort rather than a population-representative sample of all patients with HCC. Patients treated in tertiary centers may differ systematically from those in county hospitals, community clinics, or primary care settings with respect to disease severity, access to specialists, exposure to health information, and psychosocial support resources. Moreover, although the sample included participants from different regions, the hospital-based recruitment strategy may still underrepresent patients from less-resourced rural settings or those with limited access to higher-level oncology care. Future research should use stratified or multistage sampling across different healthcare levels and geographic contexts to improve representativeness and external validity.

Third, all major variables were assessed by self-report. Although Harman’s single-factor test suggested that common method bias was unlikely to be severe, shared method variance, recall bias, and social desirability bias cannot be fully ruled out. This issue is particularly relevant for treatment adherence, which was measured using a self-reported scale rather than objective behavioral indicators. Future studies should triangulate self-reported adherence with more objective measures, such as pharmacy refill records, prescription possession ratios, appointment attendance, clinician-rated adherence, or electronic medication monitoring data. Fourth, the counterintuitive pattern observed for fear of progression indicates that psycho-oncological constructs may not operate in a simple linear fashion. Although the present study offers an existential and defensive-processing interpretation, the available data did not directly assess relevant mechanisms such as meaning in life, experiential avoidance, depressive withdrawal, emotion regulation, or coping style. Future studies should incorporate these variables to test competing explanations more directly and to better distinguish adaptive vigilance from defensive disengagement.

Another limitation is that several potentially important contextual and interpersonal factors were not measured in the present study. In particular, social support and trust in physicians may be highly relevant to both death anxiety and treatment adherence among patients with HCC. Social support from family members, peers, and caregivers may buffer existential distress, facilitate emotional adjustment, and provide practical assistance for attending follow-up visits, managing medication, and maintaining engagement with treatment (74, 75). Likewise, trust in physicians may influence patients’ acceptance of medical recommendations, perceived treatment credibility, communication quality, and willingness to sustain long-term adherence under uncertainty (76). Because these variables were not included in the current dataset, residual confounding cannot be excluded, and the observed associations between psychosocial factors and profile membership should be interpreted with caution. Future research should incorporate broader interpersonal and healthcare-system variables, including social support, physician trust, family functioning, and patient–provider communication, to build a more comprehensive explanatory model of psycho-behavioral profile formation in HCC.

## 5 Conclusions

Using latent profile analysis, this study identified three qualitatively distinct joint profiles of death anxiety and treatment adherence in patients with HCC: low death anxiety–high adherence, moderate death anxiety–moderate adherence, and high death anxiety–low adherence. These findings support the presence of substantial psycho-behavioral heterogeneity within this population. Health literacy and fear of progression emerged as important correlates of profile membership, while marital status, residence, and cancer stage also showed differential associations across profiles. In particular, the high death anxiety–low adherence profile may represent a clinically meaningful high-risk subgroup for targeted screening and supportive intervention. At the same time, the findings regarding fear of progression suggest that illness-focused worry and existential death-related distress are related but distinct constructs that may diverge across subgroups. Because the study was cross-sectional and based on a tertiary-hospital convenience sample with self-reported measures, the results should be interpreted cautiously and verified in longitudinal, multi-level, and methodologically multimodal research.

## Data Availability

The raw data supporting the conclusions of this article will be made available by the authors, without undue reservation.

## References

[1] Singal AG Kanwal F Llovet JM . Global trends in hepatocellular carcinoma epidemiology: implications for screening, prevention and therapy. Nat Rev Clin Oncol. (2023) 20:864–84. doi: 10.1038/s41571-023-00825-3. PMID: 37884736

[2] Chan SL Sun HC Xu Y Zeng H El-Serag HB Lee JM . The Lancet Commission on addressing the global hepatocellular carcinoma burden: comprehensive strategies from prevention to treatment. Lancet. (2025) 406:731–78. doi: 10.1016/s0140-6736(25)01042-6. PMID: 40744051

[3] Tang K Han L Li J Li K . Machine learning-driven comprehensive profiling of tumor heterogeneity and sialylation in hepatocellular carcinoma. NPJ Precis Oncol. (2025). doi: 10.1038/s41698-025-01213-z. PMID: 41408439 PMC12783234

[4] Tey SK Wong SWK Chan JYT Mao X Ng TH Yeung CLS . Patient pIgR-enriched extracellular vesicles drive cancer stemness, tumorigenesis and metastasis in hepatocellular carcinoma. J Hepatol. (2022) 76:883–95. doi: 10.1016/j.jhep.2021.12.005. PMID: 34922977

[5] Pang Q Luo W Chen S Zhou H Zhang R Li Y . Efficacy and safety of D-TACE followed by D-RFA for unresectable large hepatocellular carcinoma. Front Oncol. (2025) 15:1530951. doi: 10.3389/fonc.2025.1530951. PMID: 40792272 PMC12336487

[6] Xiong T Zhang Y Liu X Leng K Li L Liu C . Multi-omics integrated analysis identifies key biomarkers for hepatocellular carcinoma. Med (Balt). (2025) 104:e44642. doi: 10.1097/md.0000000000044642. PMID: 40988212 PMC12459559

[7] Alawyia B Constantinou C . Hepatocellular carcinoma: a narrative review on current knowledge and future prospects. Curr Treat Options Oncol. (2023) 24:711–24. doi: 10.1007/s11864-023-01098-9. PMID: 37103744

[8] Graf J Stengel A . Psychological burden and psycho-oncological interventions for patients with hepatobiliary cancers-a systematic review. Front Psychol. (2021) 12:662777. doi: 10.3389/fpsyg.2021.662777. PMID: 34025526 PMC8131509

[9] Furer P Walker JR . Death anxiety: a cognitive-behavioral approach. J Cogn Psychother. (2008) 22. doi: 10.1891/0889-8391.22.2.167. PMID: 41472467

[10] Pashak TJ Nelson OM Tunstull MD Vanderstelt BH Nichols DP Hitt JM . Embrace subjectivity: existentially-informed clinical psychological science, practice, and teaching. Clin Psychol. (2023) 27:4–21. doi: 10.1080/13284207.2022.2108695. PMID: 41909888

[11] Reed DE Williamson RE Wickham RE . Memento mori: understanding existential anxiety through the existential pathway model. J Theor Soc Psychol. (2021) 5:14–25. doi: 10.1002/jts5.79. PMID: 41925066

[12] Sharpe L Curran L Butow P Thewes B . Fear of cancer recurrence and death anxiety. Psychooncology. (2018) 27:2559–65. doi: 10.1002/pon.4783. PMID: 29843188

[13] Baldassarre G I LdlS Vallette FM . Death-ision: the link between cellular resilience and cancer resistance to treatments. Mol Cancer. (2025) 24:144. doi: 10.1186/s12943-025-02339-1. PMID: 40375296 PMC12080166

[14] Tan DJH Quek SXZ Yong JN Suresh A Koh KXM Lim WH . Global prevalence of depression and anxiety in patients with hepatocellular carcinoma: systematic review and meta-analysis. Clin Mol Hepatol. (2022) 28:864–75. doi: 10.3350/cmh.2022.0136. PMID: 36263668 PMC9597225

[15] Zhu Y Liu Y Li K . Comment on ‘Correlates of death anxiety for patients with cancer: a systematic review and meta-analysis’. J Clin Nurs. (2024) 33:2799–800. doi: 10.1111/jocn.17094. PMID: 38500006

[16] Madfes IS . Death anxiety and related characteristics among hospice and nonhospice nurses. In: California School of Professional Psychology-Berkeley/Alameda. California (1990).

[17] Thummak S Uppor W Wannarit LO . Patient compliance: a concept analysis. Belitung Nurs J. (2023) 9:421–7. doi: 10.33546/bnj.2807. PMID: 37901377 PMC10600712

[18] Piña IL Di Palo KE Brown MT Choudhry NK Cvengros J Whalen D . Medication adherence: importance, issues and policy: a policy statement from the American Heart Association. Prog Cardiovasc Dis. (2021) 64:111–20. doi: 10.1016/j.pcad.2020.08.003 32800791

[19] Sutanto H Adytia GJ Elisa E Maimunah U . Advances in transarterial chemoembolization for hepatocellular carcinoma: integration with systemic therapies and emerging treatment strategies. Cancer Pathog Ther. (2026) 4:1–13. doi: 10.1016/j.cpt.2025.04.004. PMID: 41280241 PMC12639617

[20] Kinsey E Lee HM . Management of hepatocellular carcinoma in 2024: the multidisciplinary paradigm in an evolving treatment landscape. Cancers (Basel). (2024) 16(3):666. doi: 10.3390/cancers16030666. PMID: 38339417 PMC10854554

[21] Maddox TM Januzzi JL Allen LA Breathett K Brouse S Butler J . 2024 ACC expert consensus decision pathway for treatment of heart failure with reduced ejection fraction: a report of the American College of cardiology solution set oversight committee. J Am Coll Cardiol. (2024) 83:1444–88. doi: 10.1016/j.jacc.2023.12.024 38466244

[22] Xu Y Zheng X Li Y Ye X Cheng H Wang H . Exploring patient medication adherence and data mining methods in clinical big data: a contemporary review. J Evid Based Med. (2023) 16:342–75. doi: 10.1111/jebm.12548. PMID: 37718729

[23] Shukla A Patkar S Sundaram S Shah SR Ingle M Gupta A . Clinical profile, patterns of care & adherence to guidelines in patients with hepatocellular carcinoma: prospective multi-center study. J Clin Exp Hepatol. (2022) 12:1463–73. doi: 10.1016/j.jceh.2022.05.006. PMID: 36340319 PMC9630010

[24] Huang DQ Singal AG Kanwal F Lampertico P Buti M Sirlin CB . Hepatocellular carcinoma surveillance - utilization, barriers and the impact of changing aetiology. Nat Rev Gastroenterol Hepatol. (2023) 20:797–809. doi: 10.1097/00001574-200205000-00008. PMID: 37537332

[25] Religioni U Barrios-Rodríguez R Requena P Borowska M Ostrowski J . Enhancing therapy adherence: impact on clinical outcomes, healthcare costs, and patient quality of life. Med (Kaunas). (2025) 61(1):153. doi: 10.3390/medicina61010153. PMID: 39859135 PMC11766829

[26] Lustberg MB Kuderer NM Desai A Bergerot C Lyman GH . Mitigating long-term and delayed adverse events associated with cancer treatment: implications for survivorship. Nat Rev Clin Oncol. (2023) 20:527–42. doi: 10.1038/s41571-023-00776-9. PMID: 37231127 PMC10211308

[27] Pyszczynski T Greenberg J Solomon S . A dual-process model of defense against conscious and unconscious death-related thoughts: an extension of terror management theory. psychol Rev. (1999) 106:835. doi: 10.4324/9781315641393-1. PMID: 10560330

[28] Kozlowska K Walker P McLean L Carrive P . Fear and the defense cascade: clinical implications and management. Harv Rev Psychiatry. (2015) 23:263–87. doi: 10.1097/HRP.0000000000000065 PMC4495877 26062169

[29] Yang M Barker K Goodman D Park HL . Effective risk communication to promote behavioral change in patients at elevated risk for breast cancer based on the Health Belief Model. Breast J. (2018) 24:1097–8. doi: 10.1111/tbj.13086. PMID: 30079616

[30] Lo SW Chair SY Lee FK . Factors associated with health-promoting behavior of people with or at high risk of metabolic syndrome: based on the health belief model. Appl Nurs Res. (2015) 28:197–201. doi: 10.1016/j.apnr.2014.11.001. PMID: 25540911

[31] Sardessai-Nadkarni AA Street RL . Understanding the pathways linking patient-centered communication to cancer survivors’ emotional health: examining the mediating roles of self-efficacy and cognitive reappraisal. J Cancer Surviv. (2023) 17:1266–75. doi: 10.1007/s11764-022-01170-7. PMID: 35167049

[32] Gabriel AS Campbell JT Djurdjevic E Johnson RE Rosen CC . Fuzzy profiles: comparing and contrasting latent profile analysis and fuzzy set qualitative comparative analysis for person-centered research. Organizat Res Methods. (2018) 21:877–904. doi: 10.1177/1094428117752466

[33] Morin AJ Meyer JP Creusier J Biétry F . Multiple-group analysis of similarity in latent profile solutions. Organizat Res Methods. (2016) 19:231–54. doi: 10.1177/1094428115621148. PMID: 41930703

[34] Ye ZJ Zhang Z Tang Y Liang J Sun Z Hu GY . Resilience patterns and transitions in the be resilient to breast cancer trial: an exploratory latent profile transition analysis. Psycho‐Oncology. (2021) 30:901–9. doi: 10.1002/pon.5668. PMID: 33689199

[35] Hussain I Cakir O Ozdemir B . Studying internet addiction profile of university students with latent class analysis. Educ Inf Technol. (2020) 25:4937–59. doi: 10.1007/s10639-020-10203-6. PMID: 41933263

[36] Tang Q Wang Y Zhang C Zhao Y Zhou X Huang J . Profiles and influencing factors of work–family balance among nurses in China: a cross‐sectional study based on latent profile analysis. J Nurs Manage. (2025) 2025:8556545. doi: 10.1155/jonm/8556545. PMID: 40223892 PMC11919027

[37] Chaudhary K Poirion OB Lu L Garmire LX . Deep learning-based multi-omics integration robustly predicts survival in liver cancer. Clin Cancer Res. (2018) 24:1248–59. doi: 10.1158/1078-0432.ccr-17-0853. PMID: 28982688 PMC6050171

[38] Wang Z Zhou G Cao R Zhang G Zhang Y Xiao M . Harnessing multi-omics and artificial intelligence: revolutionizing prognosis and treatment in hepatocellular carcinoma. Front Immunol. (2025) 16:1592259. doi: 10.3389/fimmu.2025.1592259. PMID: 40771801 PMC12325060

[39] Shimada S Mogushi K Akiyama Y Furuyama T Watanabe S Ogura T . Comprehensive molecular and immunological characterization of hepatocellular carcinoma. EBioMedicine. (2019) 40:457–70. doi: 10.1016/j.ebiom.2018.12.058. PMID: 30598371 PMC6412165

[40] Jie B Feng ZZ Qiu Y Zhang YQ . Association between socio-demographic factors, coping style, illness perceptions and preference for disclosure/nondisclosure of diagnosis in Chinese patients with hepatocellular carcinoma. J Health Psychol. (2019) 24:1473–83. doi: 10.1177/1359105317707258. PMID: 28810462

[41] Han J Liu JE Qiu H Nie ZH Su YL . Illness cognitions and the associated socio-demographic and clinical factors in Chinese women with breast cancer. Eur J Oncol Nurs. (2018) 32:33–9. doi: 10.1016/j.ejon.2017.11.005. PMID: 29353630

[42] Yi Z Xu S Zhang P . Attitudes toward end-of-life care and preferred death locations: a latent profile analysis. Death Stud. (2025), 1–13. doi: 10.1080/07481187.2025.2491584. PMID: 40241620

[43] Kaspar R Schilling OK Diehl M Gerstorf D Rupprecht FS Sabatini S . Differences in self-perceptions of aging across the adult lifespan: the sample case of awareness of age-related gains and losses. Psychol Aging. (2023) 38:824–36. doi: 10.1037/pag0000783. PMID: 37917453

[44] Paccoud I Baumann M Le Bihan E Pétré B Breinbauer M Böhme P . Socioeconomic and behavioural factors associated with access to and use of personal health records. BMC Med Inform Decis Mak. (2021) 21:18. doi: 10.1186/s12911-020-01383-9. PMID: 33435970 PMC7805047

[45] Bahçecioğlu Turan G Türkben Polat H . The effects of illness perception on death anxiety and satisfaction with life in patients with advanced gastrointestinal cancer. Palliat Supp Care. (2024) 22:360–6. doi: 10.1017/s1478951523001244. PMID: 37620999

[46] Yang S Yan C Li J Feng Y Hu H Li Y . The death education needs of patients with advanced cancer: a qualitative research. BMC Palliat Care. (2024) 23:259. doi: 10.1186/s12904-024-01540-1. PMID: 39516770 PMC11545552

[47] Antoni MH Moreno PI Penedo FJ . Stress management interventions to facilitate psychological and physiological adaptation and optimal health outcomes in cancer patients and survivors. Annu Rev Psychol. (2023) 74:423–55. doi: 10.1146/annurev-psych-030122-124119. PMID: 35961041 PMC10358426

[48] Seah THS Coifman KG . Emotion differentiation and behavioral dysregulation in clinical and nonclinical samples: a meta-analysis. Emotion. (2022) 22:1686–97. doi: 10.1037/emo0000968. PMID: 34264705

[49] Bonanno GA Chen S Galatzer-Levy IR . Resilience to potential trauma and adversity through regulatory flexibility. Nat Rev Psychol. (2023) 2:663–75. doi: 10.1038/s44159-023-00233-5. PMID: 41933181

[50] Sørensen K Levin-Zamir D Duong TV Okan O Brasil VV Nutbeam D . Building health literacy system capacity: a framework for health literate systems. Health Promot Int. (2021) 36:i13–23. doi: 10.1093/heapro/daab153 PMC8672927 34897445

[51] Schulz PJ Pessina A Hartung U Petrocchi S . Effects of objective and subjective health literacy on patients’ accurate judgment of health information and decision-making ability: Survey study. J Med Internet Res. (2021) 23:e20457. doi: 10.2196/20457. PMID: 33475519 PMC7861996

[52] Lee H Chu HS . Moderating effect of health literacy on the relationship between diabetes self-management education and self-care monitoring activities among individuals with type 2 diabetes mellitus. BMC Public Health. (2025) 25:2530. doi: 10.1186/s12889-025-23765-2. PMID: 40702477 PMC12285151

[53] Fitzpatrick PJ . Improving health literacy using the power of digital communications to achieve better health outcomes for patients and practitioners. Front Digit Health. (2023) 5:1264780. doi: 10.3389/fdgth.2023.1264780. PMID: 38046643 PMC10693297

[54] Amalraj S Starkweather C Nguyen C Naeim A . Health literacy, communication, and treatment decision-making in older cancer patients. Oncol (Williston Park). (2009) 23:369–75. 19476267

[55] Herschbach P Dinkel A . Fear of progression. Psycho-oncology. (2013), 11–29. doi: 10.1007/978-3-642-40187-9_2. PMID: 24305766

[56] Jo E Bae KR . From fear to adaptation: The journey of patients with liver cancer living with the fear of cancer recurrence. Curr Oncol. (2025) 32:687. doi: 10.3390/curroncol32120687. PMID: 41440215 PMC12731318

[57] Tomer A Eliason G . Toward a comprehensive model of death anxiety. Death Stud. (1996) 20:343–65. doi: 10.1080/07481189608252787. PMID: 10160570

[58] Tein J-Y Coxe S Cham H . Statistical power to detect the correct number of classes in latent profile analysis. Struct Eq Model: A Multidiscip J. (2013) 20:640–57. doi: 10.1080/10705511.2013.824781. PMID: 24489457 PMC3904803

[59] Nylund KL Asparouhov T Muthén BO . Deciding on the number of classes in latent class analysis and growth mixture modeling: A Monte Carlo simulation study. Struct Eq Model: A Multidiscip J. (2007) 14:535–69. doi: 10.1080/10705510701575396. PMID: 41909888

[60] Cai W Tang YL Wu S Li H . Scale of death anxiety (SDA): Development and validation. Front Psychol. (2017) 8:858. doi: 10.3389/fpsyg.2017.00858. PMID: 28620327 PMC5449485

[61] Dong A Zhang H Ai F Kong L Lu T Zheng C . Chinese version of the death anxiety beliefs and behaviours scale: Psychometric properties based on CTT and IRT. Geriatr Nurs. (2024) 60:207–14. doi: 10.1016/j.gerinurse.2024.09.005. PMID: 39270407

[62] Cai W Cai H Li H . Why do humans pursue higher social class? Death anxiety matters. Death Stud. (2022) 46:434–41. doi: 10.1080/07481187.2020.1740828. PMID: 32180539

[63] Wang Y Wang X Wang X Naqvi AA Zhang Q Zang X . Translation and validation of the Chinese version of the general medication adherence scale (GMAS) in patients with chronic illness. Curr Med Res Opin. (2021) 37:829–37. doi: 10.1080/03007995.2021.1901680. PMID: 33719815

[64] Cao W Wang J Wang Y Hassan II Kadir AA . mHealth app to improve medication adherence among older adult stroke survivors: Development and usability study. Digit Health. (2024) 10:20552076241236291. doi: 10.1177/20552076241236291. PMID: 38465293 PMC10921861

[65] Fang F Zhai X Fan X Bai R Bao S Ma Y . Effectiveness of improving medication adherence and lung function for patients with chronic obstructive pulmonary disease (COPD) in pharmacists-managed outpatient clinic (PMC): A prospective before-and-after intervention study. J Eval Clin Pract. (2025) 31:e70218. doi: 10.1111/jep.70218. PMID: 40717499

[66] Sun X Lv K Wang F Ge P Niu Y Yu W . Validity and reliability of the Chinese version of the health literacy scale short-form in the Chinese population. BMC Public Health. (2023) 23:385. doi: 10.1186/s12889-023-15237-2. PMID: 36823591 PMC9951431

[67] Liu G Qi F Gao Q Huo L Jia X Wang R . The relationship between health literacy and problematic internet use in Chinese college students: The mediating effect of subject well-being and moderating effect of social support. J Affect Disord. (2024) 362:877–84. doi: 10.1016/j.jad.2024.07.038. PMID: 39009310

[68] Shi B Zhang J Wang H Rao X Sun Y Cui W . Latent profile analysis and related factors of colorectal cancer knowledge and health beliefs and their associations with screening behavior and intention among urban populations in China. Cancer Nurs. (2025) 2024:10.1097. doi: 10.1097/ncc.0000000000001479. PMID: 39982942

[69] Mahendran R Liu J Kuparasundram S Griva K . Validation of the English and simplified Mandarin versions of the fear of progression questionnaire - short form in Chinese cancer survivors. BMC Psychol. (2020) 8:10. doi: 10.1186/s40359-020-0374-0. PMID: 32005291 PMC6995061

[70] Akoglu H . User’s guide to correlation coefficients. Turk J Emergency Med. (2018) 18:91–3. doi: 10.1016/j.tjem.2018.08.001. PMID: 30191186 PMC6107969

[71] Llovet JM Brú C Bruix J . Prognosis of hepatocellular carcinoma: The BCLC staging classification. Semin Liver Dis. (1999) 19:329–38. doi: 10.1055/s-2007-1007122. PMID: 10518312

[72] Kline RB . Principles and practice of structural equation modeling. New York: Guilford Publications (2023).

[73] Li QP Mak YW Loke AY . Spouses’ experience of caregiving for cancer patients: A literature review. Int Nurs Rev. (2013) 60:178–87. doi: 10.1111/inr.12000. PMID: 23692000

[74] McAndrew NS Gray TF Wallace L Calkins K Guttormson J Harding ES . Existential distress in family caregivers: Scoping review of meaning-making interventions. BMJ Supp Palliat Care. (2024) 13:e676–85. doi: 10.1136/spcare-2023-004448. PMID: 37604657 PMC11040498

[75] Salifu Y Ekpor E Bayuo J Akyirem S Nkhoma K . Patients’ and caregivers’ experiences of familial and social support in resource-poor settings: A systematically constructed review and meta-synthesis. Palliat Care Soc Pract. (2025) 19:26323524251349840. doi: 10.1177/26323524251349840. PMID: 40584974 PMC12205196

[76] Wu D Lowry PB Zhang D Tao Y . Patient trust in physicians matters-understanding the role of a mobile patient education system and patient-physician communication in improving patient adherence behavior: Field study. J Med Internet Res. (2022) 24:e42941. doi: 10.2196/42941. PMID: 36538351 PMC9776535

